# Safety evaluation of high‐dose intake of casein‐derived peptide Met‐Lys‐Pro in healthy adults: A randomized, double‐blind, placebo‐controlled trial

**DOI:** 10.1002/fsn3.2028

**Published:** 2020-11-23

**Authors:** Naoki Yuda, Miyuki Tanaka, Masahiko Tokushima, Fumiaki Abe

**Affiliations:** ^1^ Food Ingredients and Technology Institute Morinaga Milk Industry Co., Ltd. Kanagawa Japan; ^2^ Maebashi North Hospital Gunma Japan

**Keywords:** angiotensin I‐converting enzyme, blood pressure, cognitive function, Met‐Lys‐Pro, peptide

## Abstract

Met‐Lys‐Pro (MKP) is a casein‐derived angiotensin I‐converting enzyme inhibitory peptide with the potential to cross the blood–brain barrier. It has shown preventive effects against high blood pressure (BP) and cognitive decline in animal models and human studies. MKP shows good water solubility and can be blended into a variety of foods. However, its ease of intake may contribute to the possibility of overdose. Therefore, we aimed to evaluate the safety of high‐dose intake of MKP in healthy adults by conducting a randomized controlled trial with 30 subjects. Participants were randomly allocated to the MKP (*n* = 15) or placebo (*n* = 15) group. Over 4 weeks, the MKP group received test powder containing a daily dose of 1,000 μg of MKP, five times the minimum effective dose for cognitive improvement, whereas the placebo group received dextrin powder containing no detectable MKP. No clinical problems were observed in anthropometric and BP measurements or in blood and urine parameters. No adverse events owing to MKP intake were observed. These findings suggest that consumption of MKP is safe, and that it may be applicable as a safe preventive measure against hypertension and cognitive decline in future.

## Graphical abstract

The aim of the present study was to evaluate the safety of high‐dose intake of MKP in healthy adults. No adverse events that could be associated with the study products were observed in anthropometric and BP measurements, blood and urine analysis, or medical interviews. These findings suggest that the intake of MKP is safe.

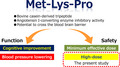

## 1 INTRODUCTION

Cognitive decline is considered a normal consequence of aging; the total number of individuals with dementia was 50 million in 2015 and is predicted to reach 82 million by 2030 and 152 million by 2050 (World Health Organization, 2019). Therefore, prevention and care for cognitive decline are issues that need to be addressed worldwide. Alzheimer's disease (AD) is the most common cause of dementia, which impairs memory, thinking skills, and behavior. The physiological changes leading to AD begin to develop decades before the earliest clinical symptoms appear, and when symptoms become clinically apparent, disease progression is too advanced for treatment (Jack et al., 2010). Therefore, current research has focused on intervention in cognitively healthy individuals at risk of developing AD to reduce its incidence and prevalence (Crous‐Bou et al., 2017).

Recently, centrally active angiotensin I‐converting enzyme (ACE) inhibitors have gained attention as a new therapy for AD (O'Caoimh et al., 2014). ACE activity in the brains of patients with AD is elevated compared with that in the brains of nondementia patients (Arregui et al., 1982; Miners et al., 2008). In addition, ACE generates elevated levels of angiotensin II in the brains of patients with AD (Savaskan et al., 2001), which promotes neurodegeneration and brain aging (Dong et al., 2011; Labandeira‐Garcia et al., 2017). Therefore, ACE inhibition represents a potential neuroprotective approach for AD. Especially, centrally active ACE inhibitors with brain‐penetrating ability that prevent the progression of neurodegeneration potentially protect against the development of AD (Fazal et al., 2017; Gao et al., 2013; O'Caoimh et al., 2014; Sink et al., 2009).

Food‐derived bioactive peptides have been considered for human health applications beyond their caloric value (Aluko, 2015; Cicero et al., 2017; Manzanares et al., 2019; Mills et al., 2011; Udenigwe & Aluko, 2012). For example, these peptides have potential antihypertensive, antioxidant, lipid‐lowering, immunomodulatory, antimicrobial, anticancer, antidiabetic, and mineral binding activities. The antihypertensive effect of ACE inhibition is one of the most studied properties of food‐derived bioactive peptides (Udenigwe & Aluko, 2012).

We previously identified the bovine casein‐derived tripeptide Met‐Lys‐Pro (MKP) as having a relatively strong ACE inhibitory activity (IC_50_ = 0.43 μM) and antihypertensive effect in spontaneously hypertensive rats (Yamada et al., 2013, 2015). In a randomized controlled trial, 100 μg of MKP daily for 12 weeks safely lowered systolic blood pressure (SBP) in humans with high‐normal blood pressure (BP) or grade 1 hypertension (Yuda et al., 2018). Furthermore, orally administered MKP is distributed in the brain and significantly attenuates cognitive decline in vivo (Min et al., 2017). In stroke‐prone spontaneously hypertensive rats, MKP administration showed neuroprotective effects by regulating cerebral circulation and corticoid secretion (Tada et al., 2020). A clinical trial for community‐dwelling adults without dementia demonstrated that daily oral intake of 200 μg of MKP over 24 weeks can improve orientation, with good tolerability and without treatment‐related adverse effects (Yuda et al., 2020). Therefore, MKP intake may be a safe preventive measure against not only hypertension but also cognitive decline.

MKP shows good water solubility and can be blended into a variety of foods; however, the ease of intake may contribute to the possibility of overdose. Therefore, we conducted a 4‐week, randomized, double‐blind, placebo‐controlled trial in healthy adults to evaluate the safety of daily intake of 1,000 μg of MKP, which is five times the minimum effective dose for cognitive improvement (Yuda et al., 2020).

## 2 MATERIALS AND METHODS

### 2.1 Subjects

Healthy adult volunteers aged ≥20 years living in Maebashi and the surrounding areas were recruited in January 2020. Exclusion criteria included a history of serious illness, treatment with medication for lifestyle‐related disease (e.g., diabetes, hypertension, dyslipidemia), digestive tract diseases or history of gastrointestinal surgery, serious allergies to medicine or food, history of drug dependence or alcoholism, participation or planned participation in other clinical studies, ineligibility owing to physician's diagnosis based on clinical laboratory test results and interview, or pregnancy, lactation, or intent to become pregnant during the study period.

The study protocol was examined and approved by the Ethical Committee of Kobuna Orthopedics Clinic (approval code: MK1911‐3). The study was conducted in accordance with the Declaration of Helsinki and the Ethical Guidelines for Medical and Health Research Involving Human Subjects (Ministry of Education, Culture, Sports, Science and Technology; Ministry of Health, Labor and Welfare, Japan). After receiving a detailed explanation of the objectives and procedures of the study, all subjects provided written informed consent and were informed that they were free to withdraw at any time without obligation. This trial was registered at the University Hospital Medical Information Network Clinical Trials Registry as UMIN000038963 prior to subject enrollment.

### 2.2 Study products

Morinaga Milk Industry (Tokyo, Japan) prepared the placebo along with the test product containing the equivalent of five times the minimum effective dose for cognitive improvement (Yuda et al., 2020). Specifically, the test powder contained 1,000 μg of MKP in 5.0 g of casein hydrolysate, and the placebo powder contained 5.0 g of dextrin with no detectable MKP. The two different products were matched for appearance.

### 2.3 Procedures

The study was designed as a randomized, double‐blind, placebo‐controlled trial, conducted at Maebashi North Hospital in Gunma, Japan between January and March 2020. As this was the first clinical trial to evaluate the safety of high‐dose intake of MKP, the sample size was determined by referring to a similar trial on food ingredient (Akazome et al., 2010). Eligible subjects were randomly allocated to receive either MKP or placebo in a 1:1 ratio by a person not directly involved in the study using computer‐generated lists of random numbers via the randomly permuted block method. Subjects, physicians, researchers assessing outcomes, and researchers conducting statistical analyses were blinded to treatment group allocation over the study duration.

This study consisted of a 2‐week preintake period, a 4‐week intake period, and a 2‐week follow‐up period. During the intake period, subjects were instructed to consume MKP or placebo powder with 150–200 ml of water daily for 4 weeks. All subjects were instructed to avoid marked alterations to diet or lifestyle and excessive drinking or eating throughout the study period. The subjects were also asked to maintain diary records of items related to study products, illness, use of medications, and hospital visits. Treatment compliance was assessed by inspecting diaries. Subjects were instructed to visit the clinic for screening 3 weeks prior to commencing the experiment, at week 0 at the beginning of the experimental period, at weeks 2 and 4 during the experiment, and at week 6 after a follow‐up period. At these visits, subjects were interviewed, anthropometric and BP measurements were made, and blood and urine samples were collected. Physicians interviewed subjects throughout the study to assess the physical condition, subjective symptoms, and any adverse events (AEs).

### 2.4 Anthropometric and BP measurements

Height was measured only during screening. Body weight (BW), SBP, diastolic BP (DBP), and pulse rate were measured at all clinic visits. BW was measured using a weighing scale with 200 kg capacity, with ranges of 0–100 kg in 100 g resolution and 100–200 kg in 200 g resolution (AD‐6207A, A&D Company, Tokyo, Japan). During BW measurement the subjects wore light clothes and no shoes. Body mass index (BMI) was calculated as BW (in kilograms) divided by the square of height (in meters). BP and pulse rate were measured using an automatic BP monitor (TM‐2656VPW, A&D Company) in a seated position.

### 2.5 Blood and urine analysis

Blood and urine analyses were performed at all clinic visits. White blood cell count, red blood cell count, hemoglobin, hematocrit, platelet count, mean corpuscular volume (MCV), mean corpuscular hemoglobin (MCH), mean corpuscular hemoglobin concentration, and leukocyte differentiation (percentage of neutrophils, lymphocytes, monocytes, eosinophils, and basophils) were recorded. Aspartate aminotransferase, alanine aminotransferase, lactic dehydrogenase, total bilirubin, alkaline phosphatase, γ‐glutamyl transpeptidase, creatine kinase (CK), fasting blood glucose, hemoglobin A1c (HbA1c), total cholesterol, low‐density lipoprotein cholesterol, high‐density lipoprotein cholesterol, triglyceride, total protein, albumin, blood urea nitrogen, creatinine, uric acid (UA), sodium, chloride, potassium, calcium, inorganic phosphorus, magnesium, and serum iron were also recorded. Urine specific gravity (USG), urine pH (U‐pH), urine protein (U‐pro), urine glucose (U‐glu), urine urobilinogen (U‐uro), urine bilirubin (U‐bil), urine ketone body (U‐ket), and occult blood reaction (OBR) were measured. Hematological examination, blood biochemical examination, and urinalysis were performed by the LSI Medience Corporation.

### 2.6 AEs and side effects

All subjects were monitored throughout the study for AEs and side effects. Safety monitoring included a questionnaire on general health and the occurrence of any health‐related events. Physicians considered the results of interviews and subjects’ diaries at weeks 0, 2, 4, and 6, and determined relationships between any AEs and ingestion of study products while remaining blinded to group allocation.

### 2.7 Statistical analysis

Statistical analysis was based on the full dataset, defined as all randomized participants receiving study treatment with at least one test result after treatment. Values are presented as means ± standard deviations. For continuous variables, statistically significant differences between the study groups were examined using Student's *t*‐test, and changes from baseline values within the same groups were analyzed using a paired *t*‐test. For categorical data, statistically significant differences between study groups were examined using Fisher's exact test. For urinalyses except for specific gravity and pH, data were coded as 0 or 1 as within or outside the reference range, respectively, then expressed as a matrix of the number of subjects and codes and analyzed using Fisher's exact test. Findings were regarded as significant at *p* < .05 according to a two‐tailed test. All statistical analyses were performed using IBM SPSS Statistics version 23 (IBM Corp.).

## 3 RESULTS

### 3.1 Subjects

**Figure 1:**
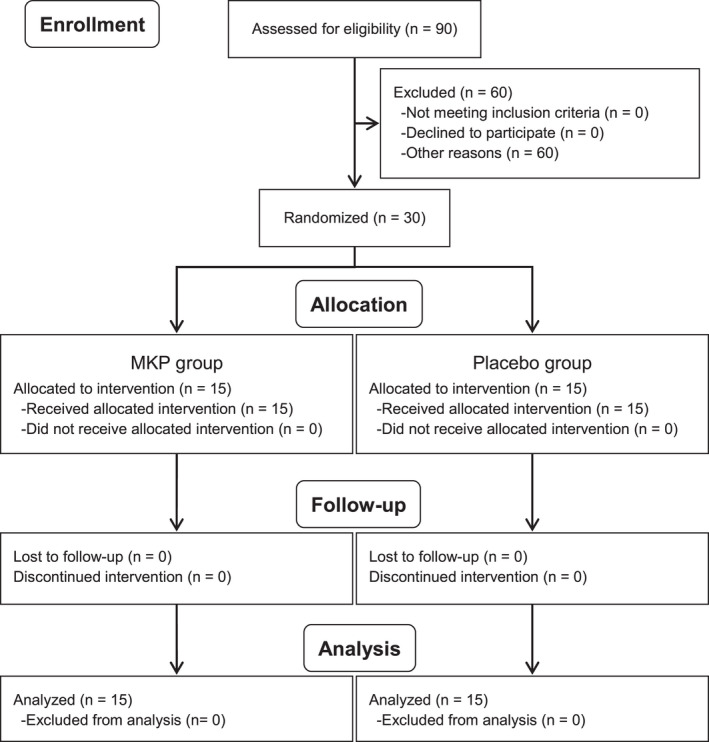
Study flow diagram. MKP, Met‐Lys‐Pro

Of a total of 90 participants screened for the study, 30 subjects (40.3 ± 12.1 years, 15 males, 15 females) were enrolled and randomly allocated into the MKP (*n* = 15) or placebo (*n* = 15) groups (Figure 1). All subjects completed the study. Table 1 shows the characteristics of the subjects at the screening period. The two groups did not differ significantly in demographic variables, including blood and urine analysis (data not shown). Demographic means of both groups were within the reference range. The treatment compliance rate of both groups was 100%.

**Table 1:** Subject characteristics at the screening period

Characteristics	MKP (*n* = 15)	Placebo (*n* = 15)
Male/Female (*n*)	7/8	8/7
Age (years) [range]	40.3 ± 12.8 [21–60]	40.3 ± 11.8 [23–62]
Height (cm) [range]	166.2 ± 4.2 [156.4–172.0]	163.8 ± 8.0 [146.5–177.0]
BW (kg) [range]	61.8 ± 7.8 [53.5–85.0]	61.5 ± 10.7 [48.0–82.1]
BMI (kg/m^2^) [range]	22.3 ± 2.5 [19.1–29.8]	22.9 ± 2.8 [19.1–28.0]
SBP (mmHg) [range]	121.8 ± 7.7 [112–138]	123.9 ± 8.0 [110–138]
DBP (mmHg) [range]	72.8 ± 7.1 [64–86]	73.6 ± 9.0 [60–87]
Pulse rate (beats/min) [range]	70.5 ± 8.2 [62–85]	68.8 ± 6.8 [58–78]

Note

Data represent number of subjects or means ± standard deviations.

Abbreviations: BMI, body mass index; BW, body weight; DBP, diastolic blood pressure; MKP, Met‐Lys‐Pro; SBP, systolic blood pressure.

### 3.2 Anthropometric and BP measurements

A summary of the anthropometric and BP measurements at baseline (week 0), during treatment (week 2 and 4), and post‐treatment (week 6) is presented in Table 2. DBP was significantly lower in the MKP group than in the placebo group at week 4 (mean difference with 95% CI: −6.20 [−11.33, −1.07] mmHg, *p* = .020). Moreover, DBP in the MKP group was significantly decreased at week 4 compared with that at week 0 (mean difference with 95% CI: −4.33 [−7.10, −1.57] mmHg, *p* = .005). The pulse rate in the placebo group was significantly increased at week 2 compared with that at week 0 (mean difference with 95% CI: 1.60 [0.01, 3.19] beats/min, *p* = .049). BW, BMI, and SBP did not significantly vary between groups or within each group. No clinical problems were noted in anthropometric or BP measurements.

**Table 2:** Summary of anthropometric and blood pressure measurements

Variables	Group	Week 0	Week 2	Week 4	Week 6
BW (kg)	MKP	61.8 ± 7.8	61.7 ± 7.9	61.7 ± 8.0	61.6 ± 8.0
	Placebo	61.5 ± 10.7	61.5 ± 10.7	61.7 ± 10.5	61.6 ± 10.5
BMI (kg/m^2^)	MKP	22.3 ± 2.5	22.3 ± 2.5	22.3 ± 2.5	22.3 ± 2.5
	Placebo	22.9 ± 2.8	22.9 ± 2.9	22.9 ± 2.8	22.9 ± 2.9
SBP (mmHg)	MKP	121.8 ± 7.7	121.7 ± 6.2	119.1 ± 7.1	119.4 ± 8.1
	Placebo	123.9 ± 8.0	124.2 ± 7.5	121.3 ± 9.8	120.9 ± 10.1
DBP (mmHg)	MKP	72.8 ± 7.1	71.2 ± 8.1	68.5 ± 7.2^**†^	70.8 ± 9.2
	Placebo	73.6 ± 9.0	74.3 ± 7.7	74.7 ± 6.5	73.7 ± 7.0
Pulse rate (beats/min)	MKP	70.5 ± 8.2	69.8 ± 7.6	69.5 ± 6.4	70.1 ± 6.6
	Placebo	68.8 ± 6.8	70.4 ± 6.7*	70.0 ± 6.5	70.1 ± 6.2

Note

Data represent means ± standard deviations.

Abbreviations: BMI, body mass index; BW, body weight; DBP, diastolic blood pressure; MKP, Met‐Lys‐Pro; SBP, systolic blood pressure.

* *p* < .05 and ^**^ *p* < .01 indicate significant difference compared with week 0 values according to paired *t*‐test; ^†^ *p* < .05 indicates significant difference compared with the placebo group according to Student's *t*‐test.

### 3.3 Blood and urine analysis

No clinical problems were apparent in the blood and urine analysis. Table 3 and 4 give summaries of the hematology test results. The hematocrit in the MKP group was significantly decreased at week 6 compared with that at week 0 (mean difference with 95% CI: −0.97 [−1.56, −0.39] %, *p* = .003). MCV in the MKP group was significantly decreased at week 2, 4, and 6 compared with that at week 0 (mean difference with 95% CI: −0.87 [−1.45, −0.28], −0.87 [−1.59, −0.15], −1.20 [−1.76, −0.64] fl, *p* = .007, *p* = .022, and *p* < .001, respectively). MCH in the MKP group was significantly decreased at week 2 compared with that at week 0 (mean difference with 95% CI: −0.19 [−0.385, −0.002] pg, *p* = .048). In the placebo group, the eosinophils/leukocytes ratio was significantly increased at week 6 compared with that at week 0 (mean difference with 95% CI: 0.52 [0.02, 1.02] %, *p* = .042). The basophils/leukocytes ratio was significantly lower in the MKP group than in the placebo group at week 2, 4, and 6 (mean difference with 95% CI: −0.22 [−0.42, −0.01], −0.21 [−0.39, −0.02], −0.26 [−0.46, −0.06] %, *p* = .034, *p* = .032, and *p* = .012, respectively). Moreover, in the placebo group, the basophils/leukocytes ratio was significantly increased at week 6 compared with that at week 0 (mean difference with 95% CI: 0.20 [0.07, 0.33] %, *p* = .005). The other blood test variables did not significantly differ between groups or within each group.

**Table 3:** Summary of hematological examination (blood cell count)

Variables	Reference range	Group	Week 0	Week 2	Week 4	Week 6
WBC (×10^2^/μl)	33–90	MKP	56.5 ± 13.3	57.3 ± 16.1	59.5 ± 16.2	58.1 ± 15.1
		Placebo	57.3 ± 17.3	57.0 ± 17.2	57.1 ± 14.7	55.9 ± 14.8
RBC (×10^4^/μl)	M: 430–570	MKP	473.1 ± 36.4	471.0 ± 37.0	474.9 ± 36.5	468.1 ± 36.8
	F: 380–500	Placebo	478.1 ± 45.1	483.3 ± 43.7	475.9 ± 45.6	476.5 ± 48.1
Hb (g/dl)	M: 13.5–17.5	MKP	14.4 ± 1.4	14.2 ± 1.4	14.4 ± 1.4	14.1 ± 1.4
	F: 11.5–15.0	Placebo	14.5 ± 1.6	14.6 ± 1.5	14.4 ± 1.5	14.5 ± 1.6
Ht (%)	M: 39.7–52.4	MKP	45.1 ± 3.9	44.5 ± 4.0	44.8 ± 3.7	44.1 ± 3.7^**^
	F: 34.8–45.0	Placebo	45.3 ± 4.1	45.7 ± 4.0	44.9 ± 3.9	44.9 ± 3.9
PLT (×10^4^/μl)	14.0–34.0	MKP	27.8 ± 5.0	27.9 ± 4.8	27.7 ± 5.0	28.0 ± 5.3
		Placebo	28.0 ± 5.8	28.4 ± 6.3	28.3 ± 6.4	28.0 ± 5.8
MCV (fl)	85–102	MKP	95.3 ± 4.4	94.4 ± 4.1^**^	94.4 ± 4.1*	94.1 ± 3.6^**^
		Placebo	94.7 ± 4.6	94.7 ± 4.1	94.4 ± 3.8	94.4 ± 3.8
MCH (pg)	28.0–34.0	MKP	30.3 ± 1.6	30.1 ± 1.4*	30.2 ± 1.7	30.1 ± 1.6
		Placebo	30.4 ± 1.4	30.2 ± 1.5	30.2 ± 1.4	30.4 ± 1.4
MCHC (%)	30.2–35.1	MKP	31.9 ± 0.8	31.9 ± 0.7	32.0 ± 0.9	32.0 ± 0.9
		Placebo	32.1 ± 1.0	31.9 ± 0.9	32.0 ± 0.8	32.2 ± 0.9

Note

Data represent means ± standard deviations.

Abbreviations: F, female; Hb, hemoglobin; Ht, hematocrit; M, male; MCH, mean corpuscular hemoglobin; MCHC, mean corpuscular hemoglobin concentration; MCV, mean corpuscular volume; MKP, Met‐Lys‐Pro; PLT, platelet count; RBC, red blood cell count; WBC, white blood cell count.

* *p* < .05 and ^**^ *p* < .01 indicate significant difference compared with week 0 values according to paired *t*‐test.

**Table 4:** Summary of hematological examination (differential leukocyte ratio)

Variables	Reference range	Group	Week 0	Week 2	Week 4	Week 6
Neut (%)	40.0–75.0	MKP	54.0 ± 9.6	56.7 ± 5.2	56.4 ± 7.2	56.7 ± 7.8
		Placebo	54.2 ± 11.0	55.0 ± 8.5	53.6 ± 4.6	52.5 ± 6.8
Lympho (%)	18.0–49.0	MKP	36.7 ± 9.8	34.6 ± 5.9	34.4 ± 7.6	34.4 ± 7.4
		Placebo	36.7 ± 11.0	36.0 ± 7.7	37.4 ± 4.9	37.8 ± 6.8
Mono (%)	2.0–10.0	MKP	5.8 ± 1.3	5.7 ± 1.8	5.8 ± 1.3	5.6 ± 1.3
		Placebo	5.8 ± 1.2	6.0 ± 1.4	5.5 ± 1.5	5.7 ± 1.1
Eosino (%)	0.0–8.0	MKP	3.0 ± 1.9	2.5 ± 1.2	2.9 ± 2.2	2.8 ± 1.9
		Placebo	2.7 ± 0.8	2.3 ± 0.9	2.9 ± 0.9	3.2 ± 1.2*
Baso (%)	0.0–2.0	MKP	0.5 ± 0.3	0.4 ± 0.2^†^	0.5 ± 0.3^†^	0.5 ± 0.3^†^
		Placebo	0.6 ± 0.2	0.6 ± 0.3	0.7 ± 0.2	0.8 ± 0.3^**^

Note

Data represent means ± standard deviations.

Abbreviations: Baso, basophils/leukocytes; Eosino, eosinophils/leukocytes; F, female; Lympho, lymphocytes/leukocytes; M, male; MKP, Met‐Lys‐Pro; Mono, monocytes/leukocytes; Neut, neutrophils/leukocytes.

* *p* < .05 and ^**^ *p* < .01 indicate significant difference compared with week 0 values according to paired *t*‐test; ^†^ *p* < .05 indicates significant difference compared with the placebo group according to Student's *t*‐test.

Table 5, 6, and 7 show summaries of the blood biochemistry analysis. At week 0, the mean CK in the placebo group exceeded the reference range, because the value of one male in the placebo group was abnormal (2,296 U/L). A re‐examination was performed one week later, and as this yielded a normal value (154 U/L), the subject was allowed to continue the experiment. CK in the MKP group was significantly increased at week 2 compared with that at week 0 (mean difference with 95% CI: 23.73 [3.62, 43.9] U/L, *p* = .024). UA in the MKP group was significantly decreased at week 4 compared with that at week 0 (mean difference with 95% CI: −0.37 [−0.71, −0.04] mg/dl, *p* = .030). HbA1c in the placebo group was significantly decreased at week 6 compared with that at week 0 (mean difference with 95% CI: −0.06 [−0.11, −0.01] %, *p* = .023). At week 6, chloride was higher in the MKP group than in the placebo group (mean difference with 95% CI: 1.67 [0.07, 3.27] mEq/L, *p* = .042). Calcium in the placebo group was significantly decreased at week 6 compared with that at week 0 (mean difference with 95% CI: −0.16 [−0.28, −0.04] mg/dl, *p* = .011). Magnesium in the MKP group was significantly decreased at week 4 compared with that at week 0 (mean difference with 95% CI: −0.16 [−0.28, −0.04] mg/dl, *p* = .028). Other blood biochemistry variables did not significantly differ between groups or within each group.

**Table 5:** Summary of blood biochemistry (proteins, pigment, and enzymes)

Variables	Reference range	Group	Week 0	Week 2	Week 4	Week 6
TP (g/dl)	6.7–8.3	MKP	7.2 ± 0.5	7.2 ± 0.4	7.1 ± 0.5	7.1 ± 0.4
		Placebo	7.2 ± 0.4	7.3 ± 0.4	7.1 ± 0.3	7.1 ± 0.3
ALB (g/dl)	3.8–5.2	MKP	4.4 ± 0.3	4.4 ± 0.3	4.4 ± 0.3	4.4 ± 0.3
		Placebo	4.5 ± 0.2	4.5 ± 0.2	4.4 ± 0.3	4.5 ± 0.2
TB (mg/dl)	0.2–1.2	MKP	0.8 ± 0.3	0.8 ± 0.3	0.8 ± 0.2	0.8 ± 0.2
		Placebo	0.8 ± 0.3	1.0 ± 0.5	0.8 ± 0.4	1.0 ± 0.4
AST (U/L)	10–40	MKP	19.7 ± 5.6	20.6 ± 4.0	19.0 ± 5.7	19.4 ± 5.5
		Placebo	20.3 ± 11.5	18.7 ± 4.9	21.5 ± 12.5	18.5 ± 6.5
ALT (U/L)	5–45	MKP	14.0 ± 5.9	15.4 ± 5.3	14.3 ± 6.7	14.7 ± 6.5
		Placebo	15.9 ± 8.2	16.5 ± 10.2	20.5 ± 19.5	20.9 ± 22.2
LD (U/L)	120–240	MKP	173.1 ± 28.5	172.7 ± 28.9	168.5 ± 28.2	170.1 ± 27.0
		Placebo	178.5 ± 18.6	173.5 ± 23.2	176.1 ± 24.9	172.7 ± 16.3
ALP (U/L)	100–325	MKP	196.9 ± 54.0	191.9 ± 51.3	192.3 ± 48.8	193.1 ± 49.7
		Placebo	167.9 ± 34.1	167.0 ± 33.1	166.3 ± 39.8	167.8 ± 35.4
γ‐GT (U/L)	M: ≤80	MKP	22.7 ± 20.6	22.0 ± 17.7	22.2 ± 20.6	22.9 ± 19.0
	F: ≤30	Placebo	21.0 ± 9.4	21.4 ± 11.2	22.5 ± 9.6	22.3 ± 9.6
CK (U/L)	M: 60–270	MKP	89.5 ± 45.8	113.3 ± 77.4*	97.6 ± 68.8	110.7 ± 87.4
	F: 40–150	Placebo	273.4 ± 569.1	103.0 ± 25.9	173.1 ± 269.6	94.8 ± 19.4

Note

Data represent means ± standard deviations.

Abbreviations: ALB, albumin; ALP, alkaline phosphatase; ALT, alanine aminotransferase; AST, aspartate transaminase; CK, creatine kinase; F, female; LD, lactate dehydrogenase; M, male; MKP, Met‐Lys‐Pro; TB, total bilirubin; TP, total protein; γ‐GT, γ‐glutamyltransferase.

* *p* < .05 indicates significant difference compared with week 0 values according to paired *t*‐test.

**Table 6:** Summary of blood biochemistry (low molecular nitrogen compounds, carbohydrates, and lipids)

Variables	Reference range	Group	Week 0	Week 2	Week 4	Week 6
BUN (mg/dl)	8.0–20.0	MKP	12.9 ± 4.6	13.6 ± 4.1	13.4 ± 3.4	12.8 ± 3.9
		Placebo	11.8 ± 2.0	11.5 ± 1.9	11.4 ± 2.5	11.5 ± 1.8
CRE (mg/dl)	M: 0.61–1.04	MKP	0.7 ± 0.1	0.7 ± 0.2	0.7 ± 0.2	0.7 ± 0.1
	F: 0.47–0.79	Placebo	0.7 ± 0.1	0.7 ± 0.1	0.7 ± 0.1	0.7 ± 0.1
UA (mg/dl)	M: 3.8–7.0	MKP	5.1 ± 1.5	4.8 ± 1.2	4.7 ± 1.2*	5.0 ± 1.3
	F: 2.5–7.0	Placebo	5.1 ± 1.1	4.9 ± 1.2	5.0 ± 1.2	5.0 ± 1.3
FBG (mg/dl)	70–109	MKP	82.1 ± 8.6	82.7 ± 6.1	84.4 ± 8.4	80.6 ± 5.8
		Placebo	83.3 ± 8.9	83.3 ± 7.2	82.9 ± 6.9	80.9 ± 9.2
HbA1c (%)	4.6–6.2	MKP	5.3 ± 0.2	5.3 ± 0.2	5.3 ± 0.2	5.3 ± 0.2
		Placebo	5.4 ± 0.2	5.4 ± 0.3	5.3 ± 0.2	5.3 ± 0.2*
TC (mg/dl)	120–219	MKP	193.5 ± 30.5	194.4 ± 29.5	191.0 ± 34.2	190.9 ± 28.0
		Placebo	197.1 ± 20.9	203.7 ± 25.4	193.7 ± 23.2	200.9 ± 22.5
LDL‐C (mg/dl)	65–139	MKP	110.0 ± 26.0	110.4 ± 27.3	107.7 ± 30.0	109.1 ± 25.5
		Placebo	109.9 ± 20.3	117.1 ± 25.4	108.4 ± 22.5	116.2 ± 23.6
HDL‐C (mg/dl)	M: 40–85	MKP	65.7 ± 9.6	67.7 ± 10.5	66.8 ± 9.7	67.2 ± 8.8
	F: 40–95	Placebo	68.5 ± 14.1	71.5 ± 15.1	67.7 ± 13.2	71.1 ± 14.1
TG (mg/dl)	30–149	MKP	87.0 ± 39.0	80.7 ± 49.3	80.7 ± 33.0	69.8 ± 20.7
		Placebo	87.6 ± 35.3	73.7 ± 29.0	85.7 ± 31.1	73.1 ± 29.7

Note

Data represent means ± standard deviations.

Abbreviations: BUN, blood urea nitrogen; CRE, creatinine; F, female; FBG, fasting blood glucose; HbA1c, hemoglobin A1c; HDL‐C, high‐density lipoprotein cholesterol; LDL‐C, low‐density lipoprotein cholesterol; M, male; MKP, Met‐Lys‐Pro; TC, total cholesterol; TG, triglyceride; UA, uric acid.

* *p* < .05 indicates significant difference compared with week 0 values according to paired *t*‐test.

**Table 7:** Summary of blood biochemistry (electrolytes and trace element)

Variables	Reference range	Group	Week 0	Week 2	Week 4	Week 6
Na (mEq/L)	137–147	MKP	140.8 ± 2.0	140.5 ± 1.4	140.1 ± 1.2	140.2 ± 1.4
		Placebo	140.5 ± 1.4	139.8 ± 2.1	140.3 ± 1.8	139.5 ± 2.1
Cl (mEq/L)	98–108	MKP	105.1 ± 2.5	104.3 ± 1.4	104.7 ± 1.6	105.5 ± 2.0^†^
		Placebo	104.1 ± 1.9	103.1 ± 1.9	104.3 ± 2.2	103.8 ± 2.3
K (mEq/L)	3.5–5.0	MKP	4.5 ± 0.4	4.3 ± 0.3	4.4 ± 0.3	4.4 ± 0.5
		Placebo	4.3 ± 0.3	4.4 ± 0.5	4.3 ± 0.5	4.3 ± 0.3
Ca (mg/dl)	8.4–10.4	MKP	9.6 ± 0.4	9.5 ± 0.3	9.5 ± 0.4	9.5 ± 0.3
		Placebo	9.6 ± 0.2	9.6 ± 0.3	9.4 ± 0.3	9.4 ± 0.3*
IP (mg/dl)	2.5–4.5	MKP	3.6 ± 0.5	3.5 ± 0.5	3.5 ± 0.6	3.7 ± 0.5
		Placebo	3.9 ± 0.7	3.8 ± 0.5	3.8 ± 0.5	3.7 ± 0.6
Mg (mg/dl)	1.9–2.5	MKP	2.2 ± 0.1	2.1 ± 0.1	2.1 ± 0.1*	2.1 ± 0.1
		Placebo	2.1 ± 0.1	2.2 ± 0.1	2.1 ± 0.1	2.1 ± 0.1
Fe (μg/dl)	M: 50–200	MKP	106.8 ± 58.7	107.8 ± 36.1	122.9 ± 50.2	118.1 ± 26.0
	F: 40–180	Placebo	109.9 ± 48.4	139.4 ± 71.0	103.2 ± 46.3	119.7 ± 56.0

Note

Data represent means ± standard deviations. Na, sodium; Cl, chloride; K, potassium; Ca, calcium; IP, inorganic phosphorus; Mg, magnesium; Fe, serum iron; M, male; F, female; MKP, Met‐Lys‐Pro.

* *p* < .05 indicates significant difference compared with week 0 values according to paired *t*‐test.

^†^ *p* < .05 indicates significant difference compared with the placebo group according to Student's *t*‐test.

Table 8 summarizes the USG and U‐pH determinations. At week 6, the U‐pH was significantly lower in the MKP group than in the placebo group (mean difference with 95% CI: −0.57 [−1.07, −0.06], *p* = .029). Moreover, the U‐pH in the placebo group was significantly increased at week 6 compared with that at week 0 (mean difference with 95% CI: 0.57 [0.09, 1.04], *p* = .023). The U‐pH did not significantly differ at any other time point, and the USG test results did not significantly vary at all between groups or within each group. Similarly, U‐pro, U‐glu, U‐uro, U‐bil, U‐ket, and OBR did not significantly vary between groups (Table 9).

**Table 8:** Summary of urine specific gravity and urine pH

Variables	Reference range	Group	Week 0	Week 2	Week 4	Week 6
USG	1.006–1.030	MKP	1.018 ± 0.008	1.018 ± 0.007	1.017 ± 0.008	1.014 ± 0.008
		Placebo	1.015 ± 0.006	1.015 ± 0.009	1.015 ± 0.007	1.016 ± 0.008
U‐pH	5.0–7.5	MKP	6.4 ± 0.7	6.7 ± 0.7	6.5 ± 0.9	6.1 ± 0.6^†^
		Placebo	6.1 ± 0.5	6.3 ± 0.8	6.3 ± 0.8	6.6 ± 0.7*

Note

Data indicate means ± standard deviations. USG, urine specific gravity; U‐pH, urine pH; MKP, Met‐Lys‐Pro.

* *p* < .05 indicates significant difference compared with the week 0 values according to paired *t*‐test;

^†^ *p* < .05 indicates significant difference compared with the placebo group according to Student's *t*‐test.

**Table 9:** Summary of urinalysis results

Variables	Week	MKP (*n* = 15)		Placebo (*n* = 15)		*p* value
		Reference range		Reference range		
		Within	Outside	Within	Outside	
U‐pro	0	15	0	15	0	NA
	2	14	1	15	0	1.00
	4	14	1	15	0	1.00
	6	15	0	15	0	NA
U‐glu	0	15	0	15	0	NA
	2	15	0	15	0	NA
	4	15	0	15	0	NA
	6	15	0	15	0	NA
U‐uro	0	15	0	15	0	NA
	2	15	0	15	0	NA
	4	15	0	15	0	NA
	6	15	0	15	0	NA
U‐bil	0	15	0	15	0	NA
	2	15	0	15	0	NA
	4	15	0	15	0	NA
	6	15	0	15	0	NA
U‐ket	0	15	0	15	0	NA
	2	15	0	15	0	NA
	4	15	0	15	0	NA
	6	15	0	15	0	NA
OBR	0	15	0	15	0	NA
	2	14	1	14	1	1.00
	4	14	1	14	1	1.00
	6	13	2	15	0	0.48

Note

Data represent the number of subjects.

Abbreviations: MKP, Met‐Lys‐Pro; NA, not applicable; OBR, occult blood reaction; U‐bil, urine bilirubin; U‐glu, urine glucose; U‐ket, urine ketone body. *p* values were calculated using Fisher's exact test; U‐pro, urine protein; U‐uro, urine urobilinogen.

### 3.4 AEs and side effects

Nine AEs were reported by seven subjects throughout the study, with one reported by one subject in the MKP group and eight reported by six subjects in the placebo group. These included elevated serum iron (two subjects in the placebo group), muscle pain (two subjects in the placebo group), elevated CK (one subject in the MKP group and three subjects in the placebo group), and elevated total bilirubin (one subject in the placebo group). All reported AEs were mild and judged to be unrelated to intake of study products.

## 4 DISCUSSION

A number of dietary factors have preventive effects on cognitive decline and AD (Solfrizzi et al., 2011; Wu & Sun, 2016; Yurko‐Mauro et al., 2015; Zhang et al., 2016), and bioactive peptides have been demonstrated to exert preventive effects on cognitive decline in animal models and human studies (Katayama & Nakamura, 2019). MKP has the potential to improve orientation in community‐dwelling adults without dementia, with good tolerability, and no treatment‐related adverse effects during 24 weeks of treatment and for 2 weeks after treatment (Yuda et al., 2020). We conducted a randomized, double‐blind, placebo‐controlled trial to examine the safety of high‐dose intake of MKP in healthy adults and found that supplementation of 1,000 μg of MKP for 4 weeks yielded no adverse outcomes. The trial showed significant differences in some parameters, but these changes did not appear to be clinically significant. Therefore, they were not considered to be clinical safety concerns attributable to the intake of the study products.

Nine AEs were reported by seven subjects throughout the study. Elevated CK was the most common, occurring in one subject in the MKP group and three subjects in the placebo group. High levels of CK in healthy subjects may be correlated with muscle damage following physical exercise (Brancaccio et al., 2008), and all subjects with elevated CK had been playing sports or doing physical work immediately prior to testing. Therefore, the CK elevation was unlikely to be caused by the consumption of the study products. Elevated serum iron, muscle pain, and elevated total bilirubin were also reported as AEs. However, all these AEs resolved without treatment and were therefore judged to be transient and unrelated to the study. These findings suggest that there are no clinical safety concerns associated with high‐dose intake of MKP.

We previously found that the daily ingestion of 100 μg of MKP for 12 weeks is safe and effective in lowering SBP in people with high‐normal BP or grade 1 hypertension (Yuda et al., 2018). In the present study, although DBP in the MKP group was significantly lower at week 4 than that in the placebo group, there was no significant difference in SBP between the MKP and placebo groups. This may be because the previous study targeted people with high‐normal BP, defined as SBP 130–139 mmHg and/or DBP 85–89 mmHg, or grade 1 hypertension, defined as SBP 140–159 mmHg and/or DBP 90–99 mmHg. In the present study, subjects’ mean BP before intervention was not high, although some subjects with high‐normal BP were included (Table 1). The lack of effect of normotensives on BP has similarly been reported in casein hydrolysate containing Val‐Pro‐Pro and Ile‐Pro‐Pro with an ACE inhibitory effect (Ishida et al., 2011). Although further studies may be needed to evaluate the effect of high‐dose MKP on individuals with high BP, these results suggest that high‐dose intake of MKP does not affect normal BP.

Our study design was a well‐controlled trial to evaluate the safety of high‐dose intake of MKP, but several limitations should be noted. Firstly, the intervention was limited to a 4‐week period. Secondly, it is still unknown whether high‐dose intake of MKP affects the safety of individuals with disease. It may be necessary to conduct an intervention with a longer test period to evaluate the safety of high‐dose intake of MKP on adults with and without disease.

In conclusion, this randomized, double‐blind, placebo‐controlled trial assessed the safety of daily intake of 1,000 μg of MKP for 4 weeks to healthy adult subjects. No AEs that could be associated with the study products were observed in anthropometric and BP measurements, blood and urine analysis, or medical interviews. These findings suggest that the MKP intake is safe and that in future, it may be applicable as a safe preventive measure against hypertension and cognitive decline.

## CONFLICT OF INTEREST

This work was supported by the Morinaga Milk Industry Co., Ltd., Tokyo, Japan. NY, MT, and FA are employed by the Morinaga Milk Industry Co., Ltd., Tokyo, Japan. There are no other conflicts of interest.

## ETHICAL APPROVAL

The study protocol was examined and approved by the Ethical Committee of Kobuna Orthopedics Clinic (approval code: MK1911‐3).

## INFORMED CONSENT

After receiving a detailed explanation of the objectives and procedures of the study, all subjects provided written informed consent and were informed that they were free to withdraw at any time without obligation.

## References

[fsn32028-bib-0001] Akazome, Y. , Kametani, N. , Kanda, T. , Shimasaki, H. , & Kobayashi, S. (2010). Evaluation of safety of excessive intake and efficacy of long‐term intake of beverages containing apple polyphenols. Journal of Oleo Science, 59, 321–338. 10.5650/jos.59.321 20484838

[fsn32028-bib-0002] Aluko, R. E. (2015). Antihypertensive peptides from food proteins. Annual Review of Food Science and Technology, 6, 235–262. 10.1146/annurev-food-022814-015520 25884281

[fsn32028-bib-0003] Arregui, A. , Perry, E. K. , Rossor, M. , & Tomlinson, B. E. (1982). Angiotensin converting enzyme in Alzheimer's disease increased activity in caudate nucleus and cortical areas. Journal of Neurochemistry, 38, 1490–1492. 10.1111/j.1471-4159.1982.tb07930.x 6278093

[fsn32028-bib-0004] Brancaccio, P. , Maffulli, N. , Buonauro, R. , & Limongelli, F. M. (2008). Serum enzyme monitoring in sports medicine. Clinics in Sports Medicine, 27, 1–18. 10.1016/j.csm.2007.09.005 18206566

[fsn32028-bib-0005] Cicero, A. , Fogacci, F. , & Colletti, A. (2017). Potential role of bioactive peptides in prevention and treatment of chronic diseases: A narrative review. British Journal of Pharmacology, 174, 1378–1394. 27572703 10.1111/bph.13608 PMC5429326

[fsn32028-bib-0006] Crous‐Bou, M. , Minguillón, C. , Gramunt, N. , & Molinuevo, J. L. (2017). Alzheimer's disease prevention: From risk factors to early intervention. Alzheimer's Research & Therapy, 9, 71 10.1186/s13195-017-0297-z PMC5596480 28899416

[fsn32028-bib-0007] Dong, Y.‐F. , Kataoka, K. , Tokutomi, Y. , Nako, H. , Nakamura, T. , Toyama, K. , Sueta, D. , Koibuchi, N. , Yamamoto, E. , Ogawa, H. , & Kim‐Mitsuyama, S. (2011). Perindopril, a centrally active angiotensin‐converting enzyme inhibitor, prevents cognitive impairment in mouse models of Alzheimer's disease. FASEB Journal, 25, 2911–2920. 10.1096/fj.11-182873 21593435

[fsn32028-bib-0008] Fazal, K. , Perera, G. , Khondoker, M. , Howard, R. , & Stewart, R. (2017). Associations of centrally acting ACE inhibitors with cognitive decline and survival in Alzheimer's disease. BJPsych Open, 3, 158–164. 10.1192/bjpo.bp.116.004184 28713585 PMC5495996

[fsn32028-bib-0009] Gao, Y. , O'Caoimh, R. , Healy, L. , Kerins, D. M. , Eustace, J. , Guyatt, G. , Sammon, D. , & Molloy, D. W. (2013). Effects of centrally acting ACE inhibitors on the rate of cognitive decline in dementia. British Medical Journal Open, 3, e002881 10.1136/bmjopen-2013-002881 PMC3703568 23887090

[fsn32028-bib-0010] Ishida, Y. , Shibata, Y. , Fukuhara, I. , Yano, Y. , Takehara, I. , & Kaneko, K. (2011). Effect of an excess intake of casein hydrolysate containing Val‐Pro‐Pro and Ile‐Pro‐Pro in subjects with normal blood pressure, high‐normal blood pressure, or mild hypertension. Bioscience, Biotechnology, and Biochemistry, 75, 427–433. 10.1271/bbb.100560 21389626

[fsn32028-bib-0011] Jack, C. R. , Knopman, D. S. , Jagust, W. J. , Shaw, L. M. , Aisen, P. S. , Weiner, M. W. , Petersen, R. C. , & Trojanowski, J. Q. (2010). Hypothetical model of dynamic biomarkers of the Alzheimer's pathological cascade. The Lancet Neurology, 9, 119–128. 10.1016/S1474-4422(09)70299-6 20083042 PMC2819840

[fsn32028-bib-0012] Katayama, S. , & Nakamura, S. (2019). Emerging roles of bioactive peptides on brain health promotion. International Journal of Food Science & Technology, 54, 1949–1955. 10.1111/ijfs.14076

[fsn32028-bib-0013] Labandeira‐Garcia, J. L. , Rodríguez‐Perez, A. I. , Garrido‐Gil, P. , Rodriguez‐Pallares, J. , Lanciego, J. L. , & Guerra, M. J. (2017). Brain renin‐angiotensin system and microglial polarization: Implications for aging and neurodegeneration. Frontiers in Aging Neuroscience, 9, 129 10.3389/fnagi.2017.00129 28515690 PMC5413566

[fsn32028-bib-0014] Manzanares, P. , Gandía, M. , Garrigues, S. , & Marcos, J. F. (2019). Improving health‐promoting effects of food‐derived bioactive peptides through rational design and oral delivery strategies. Nutrients, 11, 2545 10.3390/nu11102545 PMC6836114 31652543

[fsn32028-bib-0015] Mills, S. , Ross, R. P. , Hill, C. , Fitzgerald, G. F. , & Stanton, C. (2011). Milk intelligence: Mining milk for bioactive substances associated with human health. International Dairy Journal, 21, 377–401. 10.1016/j.idairyj.2010.12.011

[fsn32028-bib-0016] Min, L.‐J. , Kobayashi, Y. , Mogi, M. , Tsukuda, K. , Yamada, A. , Yamauchi, K. , Abe, F. , Iwanami, J. , Xiao, J.‐Z. , & Horiuchi, M. (2017). Administration of bovine casein‐derived peptide prevents cognitive decline in Alzheimer disease model mice. PLoS One, 12, e0171515 10.1371/journal.pone.0171515 28158298 PMC5291428

[fsn32028-bib-0017] Miners, J. S. , Ashby, E. , Van Helmond, Z. , Chalmers, K. A. , Palmer, L. E. , Love, S. , & Kehoe, P. G. (2008). Angiotensin‐converting enzyme (ACE) levels and activity in Alzheimer's disease, and relationship of perivascular ACE‐1 to cerebral amyloid angiopathy. Neuropathology and Applied Neurobiology, 34, 181–193. 10.1111/j.1365-2990.2007.00885.x 17973905

[fsn32028-bib-0018] O'Caoimh, R. , Healy, L. , Gao, Y. , Svendrovski, A. , Kerins, D. M. , Eustace, J. , Kehoe, P. G. , Guyatt, G. , & Molloy, D. W. (2014). Effects of centrally acting angiotensin converting enzyme inhibitors on functional decline in patients with Alzheimer's disease. Journal of Alzheimer's Disease, 40, 595–603. 10.3233/JAD-131694 24496072

[fsn32028-bib-0019] Savaskan, E. , Hock, C. , Olivieri, G. , Bruttel, S. , Rosenberg, C. , Hulette, C. , & Müller‐Spahn, F. (2001). Cortical alterations of angiotensin converting enzyme, angiotensin II and AT1 receptor in Alzheimer’s dementia. Neurobiology of Aging, 22, 541–546. 10.1016/s0197-4580(00)00259-1 11445253

[fsn32028-bib-0020] Sink, K. M. , Leng, X. , Williamson, J. , Kritchevsky, S. B. , Yaffe, K. , Kuller, L. , Yasar, S. , Atkinson, H. , Robbins, M. , Psaty, B. , & Goff, D. C. (2009). Angiotensin‐converting enzyme inhibitors and cognitive decline in older adults with hypertension: Results from the Cardiovascular Health Study. Archives of Internal Medicine, 169, 1195–1202. 10.1001/archinternmed.2009.175 19597068 PMC2881686

[fsn32028-bib-0021] Solfrizzi, V. , Panza, F. , Frisardi, V. , Seripa, D. , Logroscino, G. , Imbimbo, B. P. , & Pilotto, A. (2011). Diet and Alzheimer's disease risk factors or prevention: The current evidence. Expert Review of Neurotherapeutics, 11, 677–708. 10.1586/ern.11.56 21539488

[fsn32028-bib-0022] Tada, A. M. , Hamezah, H. S. , Yanagisawa, D. , Morikawa, S. , & Tooyama, I. (2020). Neuroprotective effects of casein‐derived peptide Met‐Lys‐Pro (MKP) in a hypertensive model. Frontiers in Neuroscience, 14, 845 10.3389/fnins.2020.00845 32922259 PMC7457086

[fsn32028-bib-0023] Udenigwe, C. C. , & Aluko, R. E. (2012). Food protein‐derived bioactive peptides: Production, processing, and potential health benefits. Journal of Food Science, 77, R11–R24. 10.1111/j.1750-3841.2011.02455.x 22260122

[fsn32028-bib-0024] World Health Organization . (2019). Risk reduction of cognitive decline and dementia. Retrieved from: https://www.who.int/mental_health/neurology/dementia/guidelines_risk_reduction/en/ 31219687

[fsn32028-bib-0025] Wu, L. , & Sun, D. (2016). Meta‐analysis of milk consumption and the risk of cognitive disorders. Nutrients, 8, 824 10.3390/nu8120824 PMC5188477 27999380

[fsn32028-bib-0026] Yamada, A. , Sakurai, T. , Ochi, D. , Mitsuyama, E. , Yamauchi, K. , & Abe, F. (2013). Novel angiotensin I‐converting enzyme inhibitory peptide derived from bovine casein. Food Chemistry, 141, 3781–3789. 10.1016/j.foodchem.2013.06.089 23993549

[fsn32028-bib-0027] Yamada, A. , Sakurai, T. , Ochi, D. , Mitsuyama, E. , Yamauchi, K. , & Abe, F. (2015). Antihypertensive effect of the bovine casein‐derived peptide Met‐Lys‐Pro. Food Chemistry, 172, 441–446. 10.1016/j.foodchem.2014.09.098 25442576

[fsn32028-bib-0028] Yuda, N. , Tanaka, M. , Yamada, A. , Ochi, D. , Yamauchi, K. , Abe, F. , & Sakane, N. (2018). Antihypertensive effect of the casein‐derived peptide Met‐Lys‐Pro in individuals with high‐normal blood pressure or grade 1 hypertension: A randomized, double‐blind, placebo‐controlled, parallel‐group trial. Japanese Pharmacology & Therapeutics, 46, 529–537.

[fsn32028-bib-0029] Yuda, N. , Tanaka, M. , Yamauchi, K. , Abe, F. , Kakiuchi, I. , Kiyosawa, K. , & Nakamura, M. (2020). Effect of the casein‐derived peptide Met‐Lys‐Pro on cognitive function in community‐dwelling adults without dementia: A randomized, double‐blind, placebo‐controlled trial. Clinical Interventions in Aging, 15, 743–754. 10.2147/CIA.S253116 32546992 PMC7266326

[fsn32028-bib-0030] Yurko‐Mauro, K. , Alexander, D. D. , & Van Elswyk, M. E. (2015). Docosahexaenoic acid and adult memory: A systematic review and meta‐analysis. PLoS One, 10, e0120391 10.1371/journal.pone.0120391 25786262 PMC4364972

[fsn32028-bib-0031] Zhang, Y. , Chen, J. , Qiu, J. , Li, Y. , Wang, J. , & Jiao, J. (2016). Intakes of fish and polyunsaturated fatty acids and mild‐to‐severe cognitive impairment risks: A dose‐response meta‐analysis of 21 cohort studies. The American Journal of Clinical Nutrition, 103, 330–340. 10.3945/ajcn.115.124081 26718417

